# Successful implantation and live birth of a healthy boy after triple biopsy and double vitrification of oocyte-embryo-blastocyst

**DOI:** 10.1186/s40064-015-0788-y

**Published:** 2015-01-14

**Authors:** Ermanno Greco, Anil Biricik, Rocio P Cotarelo, Elisabetta Iammarone, Patrizia Rubino, Jan Tesarik, Francesco Fiorentino, Maria Giulia Minasi

**Affiliations:** Centre for Reproductive Medicine, European Hospital, Rome, Italy; Genoma, Molecular Genetics Laboratory, Rome, Italy; MAR&Gen Clinic, Molecular Assisted Reproduction and Genetics, Granada, Spain

**Keywords:** Vitrification, Polar body biopsy, Embryo biopsy, Blastocyst biopsy

## Abstract

**Introduction:**

Preimplantation genetic diagnosis and/or screening (PGD/PGS) allow the assessment of the genetic health of an embryo before transferring it into the uterus. These techniques require the removal of cellular material (polar bodies, blastomere(s) or trophectoderm cells) in order to perform the proper genetic analysis. We report the implantation and live birth outcome of a vitrified-warmed blastocyst developed after triple biopsy and double vitrification procedures at oocyte, cleavage embryo and blastocyst stage.

**Case description:**

An infertile couple, with family history of β-thalassemia, searched for IVF procedure and PGD. First polar bodies biopsy with subsequent vitrification was uninformative due to meiotic crossing-over, so oocytes were inseminated after warming. Two embryos were obtained and blastomere biopsy was performed on day 3 with inconclusive results on their genetic status. Their culture resulted in one expanded blastocyst stage on day 7 that underwent trophectoderm biopsy and vitrification. This embryo showed to be normal. It was then warmed and transferred in an artificial cycle.

**Discussion and Evaluation:**

Preconception genetic analysis by removal and analysis of the first polar body is technically possible, but the genetic information that we can obtain at this stage may be limited and the oocytes to be inseminated is not predictable. Compared to blastomere biopsy, trophectoderm biopsy has more diagnostic efficiency with respect to both chromosomal mosaicism and PCR accuracy, reducing the problems of amplification failure and allele drop out. Moreover, embryos biopsied at the cleavage stage seem to have lower implantation rate than biopsied blastocyst.

**Conclusions:**

This is the first case report of a live birth obtained from a three step biopsy and double vitrification procedures of a blastocyst. This case report seems also to suggest the harmlessness of all these procedures if carefully performed by a skilled biologist in an IVF lab with quality management system. Finally, our study highlight that blastocyst cryopreserved on day 7 have clinically important potential and embryos that not reach blastocyst stage on day 6 should not to be discharged because they may result in an ongoing pregnancy.

## Background

Preimplantation genetic diagnosis and/or screening (PGD/PGS) allow the assessment of the genetic health of an embryo before transferring it into the uterus. These techniques require the removal of cellular material in order to perform the proper genetic analysis.

Potential sources of genetic material are: first (1 PB) and second polar bodies (2 PB), day 3 embryo blastomeres, and blastocyst trophectoderm cells (Brezina et al. 2013; Greco et al. 2013). All these techniques require several laboratory manipulation procedures before embryo transfer: oocyte zona pellucida laser drilling, embryo biopsy, cryopreservation and thawing, that might affect embryo survival and its implantation potential (Scott et al. 2013a; Zhang et al. 2009).

One of the most critical point in PGD/PGS programs is assessing the right time to do the genetic analysis. In fact this issue needs careful considerations: accurate identification of the genetic status of the oocyte in case of monogenic disease especially when only the first polar body is analyzed, the possibility of mosaicism phenomenon and self correction mechanism at cleavage stage embryo, minimization of the biopsy adverse effect on the embryo (Scott et al. 2013a).

We report a normal blastocyst development and a successful live birth after sequential application of multiple potentially invasive biological micromanipulation techniques (polar body, blastomere and trophectoderm biopsy) and repeated vitrification and warming procedures.

## Case presentation

An infertile couple, with family history of β-thalassemia (Galanello and Origa 2010) searched for IVF procedure and preimplantation genetic diagnosis (PGD): a 37 year old woman and a 38 year old man, carriers of the IVS1-110 G > A and the Cod 39C > T mutations in the HBB gene (OMIM 141900), respectively. Seminal fluid examination according to the WHO recommendation (WHO 2010) showed a severe oligoastenoteratozoospermia. Ovarian reserve was evaluated combining antral follicle count (AFC), day 3 FSH and Antimullerian (AMH) dosage (Fleming et al. 2013). A written consent of the couple was obtained.

This study was approved by the Institutional Ethical Committee of the European Hospital and Genoma group. All techniques were performed according to the Helsinki declaration of 1975 and its modifications.

Controlled ovarian stimulation was performed using a long gonadotrophin-releasing hormone (GnRH) agonist suppression protocol starting on the 21^st^ day of the preceding cycle until the day of triggering; recombinant FSH administration (GonalF, MerkSerono, Italy) was started on the 3^rd^ day of the cycle. When at least 3 follicles reached 19 mm in diameter, 10.000 UI hCG (Gonasi, 10.000 UI, IBSA, Lodi, Italy) was administered by intramuscular injection (Huber et al. 2013). Thirty-six hour later, ten oocytes were retrieved through an ultrasound-guided transvaginal follicular puncture; five of them were found to be metaphase II.

Polar body analysis was chosen as the first diagnostic option for ethical reasons. Polar body biopsy consisted in opening the zona pellucida using infrared laser drilling. Using a polar body biopsy pipette, polar bodies were extracted from oocytes (Humagen, Origio, Charlostesville, VA, USA) through an hole into the perivitelline space making a gentle suction. After the biopsy oocytes were vitrified (Montag et al. 2013).

For cleavage stage, the blastomere biopsy pipette (Humagen, Origio, Charlostesville, VA, USA) was introduced inside the embryo using the same hole. After biopsy embryos were transferred to sequential culture media (Quinn’s Advantage Blastocyst medium, SAGE, CooperSurgical, Pasadena, CA, USA) (Kaser and Ginsburg 2014).

For blastocyst stage, using a combination of gently suction with blastomere aspiration pipette (COOK, Ireland) and the proper number of laser pulses (each one of 3,7 intensity) (Chang et al. 2013), around 6-8 trophectoderm cells were dissected from the expanded blastocyst. The blastocyst was then vitrified.

The biopsied cells were placed in RNase-DNase-free 0.2 ml PCR tubes using a 130 μm glass pipette (Humagen, Origio, Charlostesville, VA, USA), in the same way for all three kinds of cells biopsied and sent for genetic analysis. PCR conditions used and the strategy used for mutation analysis are described elsewhere (Fiorentino et al. 2006).

Vitrification, thawing procedure and materials were the same for oocytes, embryos and blastocyst (Cryotop and Vitrification kit, Kitazato Supply, Shizuoka, Japan) (Mukaida and Oka 2012).

**Figure 1:**
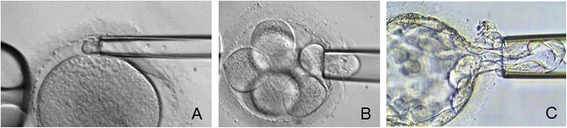
**Polar body (A), blastomere (B) and trophectoderm (C) biopsies.**

Five oocytes were submitted to PB biopsy and vitrification. Their genetic analysis revealed that one of the oocytes carried the mutation, three oocytes could not be diagnosed due to their recombinant state and one oocyte was excluded because was in telophase I stage and not suitable for biopsy. After a genetic counselling, the couple decided to proceed with the insemination of the three recombinant oocytes. The vitrified oocytes were warmed and the ICSI procedure was performed on the two survived, obtaining two 72 h cleavage stage embryos submitted to one-blastomere biopsy. Genetic analysis was uninformative, so a new biopsy was performed on the trophectoderm cells of an expanded blastocyst on day 7 immediately vitrified (Figure 1). The PCR analysis revealed that the embryo was healthy carrier for β-thalassemia and suitable for transfer.

**Figure 2:**
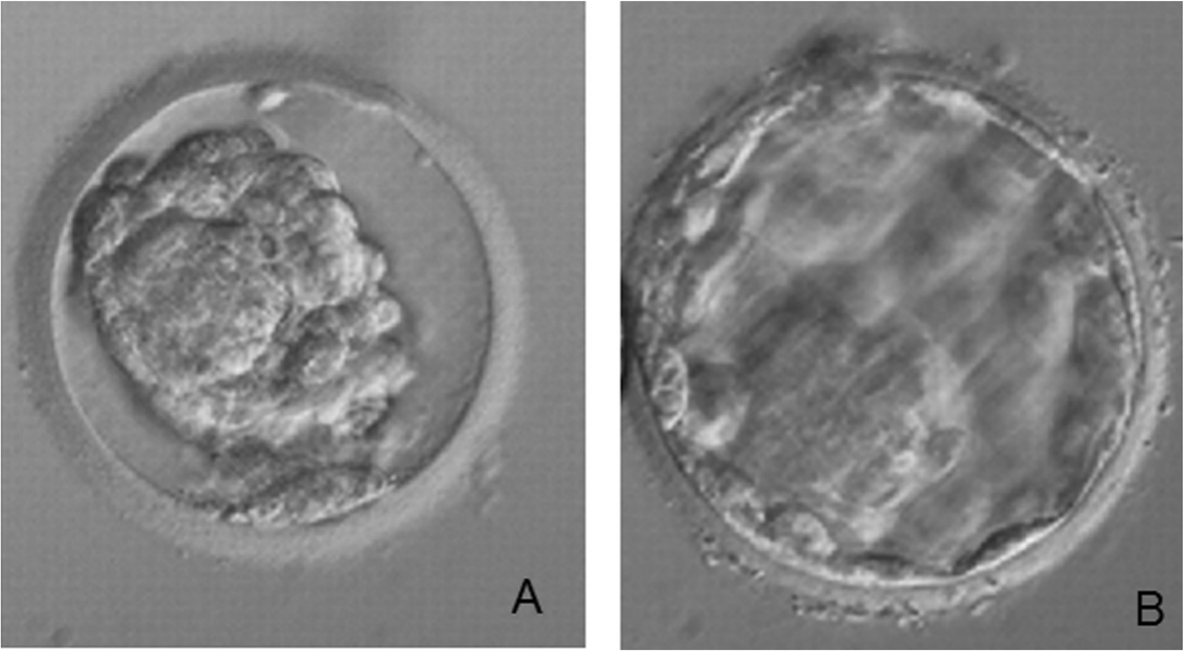
**Day-7 biopsied blastocyst wich resulted in a healthy boy, immediately after the warming (A) and two hours after incubation before the transfer (B).**

The blastocyst was warmed and transferred on the scheduled day (Figure 2). Frozen-thawed blastocyst transfer was perfomed in an artificial cycle combining gonadotrophin-releasing hormone agonist and estrogen pill (Progynova, Bayer, New Zealand limited, Auckland). Transfer was carried under ultrasound guidance when endometrium thickness reached 8 mm. Intramuscular administration of progesterone in oil (Prontogest, IBSA, Lodi, Italy) was initiated 5 days before embryo transfer (Hill et al. 2010).

A positive β-HCG was obtained 11 days after embryo transfer and fetal heart beat was confirmed by ultrasound observation 7 weeks after. The patient delivered an healthy boy weighting 3.900 g at 40 weeks of gestation. No neonatal problems have been reported.

## Discussion

This is the first case report of a live birth obtained from a three step biopsy and double vitrification procedures of a blastocyst. Many biological and clinical considerations can be drawn from this case. Preconception genetic analysis by removal and analysis of the first polar body is technically possible, but the genetic information that we can obtain at this stage may be limited and the oocytes to be inseminated is not predictable. Theoretically, the biopsy and genetic analysis of the first polar body is likely to have some advantages respect to preimplantation diagnosis performed on the blastomere or trophectoderm cells. Biopsy of the oocyte prior to fertilization is ethically accepted since it does not require manipulation of the pre-embryo. Our study confirms that aspiration of the first polar body seems to have no detrimental effects on fertilization or quality of the embryonic development until to the blastocyst stage because it is not involved in all these processes (Macas et al. 2011). Different studies, on the contrary, showed that day 3 embryos that undergone blastomere biopsy had more fragmentation and showed a slower cell division if compared with unbiopsied controls, but no definitive conclusion on the safety of PB biopsy with respect to the embryo implantation potential is available at present because all the published studies are underpowered (Montag et al. 2013; Levin et al. 2012). Biological availability of oocytes for first polar body biopsy can also be reduced at methaphase II stage. In fact, recent investigations have demonstrated that some oocytes showing a first PB may still be in telophase I stage due to the presence of a connective spindle strand between the first PB and the oocyte, as occurred in the present case (Montag et al. 2006). Often vitrification of the biopsied oocytes is necessary if the genetic analysis needs time because their fertilization rate decrease if too much time intercourses between the oocytes denudation and intracytoplasmic sperm injection (Patrat et al. 2012).

Preconception diagnosis by the removal and PCR analysis of the first polar body has been successfully applied to many genetic diseases, including sickle cell anaemia (Saiki et al. 1985), *a-*thalassaemia (Saiki et al. 1985; Chehab et al. 1987), β-thalassaemia (Saiki et al. 1988), cystic fibrosis (Kerem et al. 1989), Duchenne's muscular dystrophy (Speer et al. 1989) and Tay Sach's disease (Myerowitz 1988; Myerowitz and Costigan 1988) but the possibility of crossing over between homologous chromosomes must be considered. If this occurs, the primary oocytes will be heterozygous for the abnormal gene and the genotype of the oocyte is not correctly predictable. The probability of a crossing over occurring between any gene and the centromere is a function of the distance of the gene from the centromere. For telomeric genes, crossing over occurs with sufficient frequency that the genes segregate independently from the centromere. For telomeric genes the prediction is that one quarter of aspirated polar bodies will give the correct information and consequently only one quarter of the oocytes will be suitable for the ICSI procedure. For genes situated close to the telomere, the percentage of meiosis with crossing over between the locus and the centromere will be lower (Verlinsky et al. 1990). Our results are agree with others studies in which the percentage of recombinant oocytes for beta thalassemia gene was very high (Verlinsky et al. 1997; Fiorentino et al. 2008). In these cases biopsy of both polar bodies or biopsy of blastomere at cleavage stage, if the analysis of paternally derived mutations is needed, is necessary to give complete genetic information (Kuliev and Rechitsky 2011).

Allele drop out (ADO) and amplification failure of both alleles are well known phenomena in PCR on single cells. This can depend on several reasons: the cell type employed for the PCR (Rechitsky et al. 1998), genes and markers (Dreesen et al. 1996), lysis and PCR conditions (Levinson et al. 1992; Gitlin et al. 1996; Ray et al. 1996).

Trophectoderm biopsy has more diagnostic efficiency with respect to both chromosomal mosaicism and PCR accuracy, reducing the problems of amplification failure and allele drop out (Chang et al. 2013; Yang et al. 2013). Moreover we have to consider the effect of biopsy on implantation at cleavage stage embryos. A recent randomized controlled trial demonstrated that embryos biopsied at the cleavage stage have lower implantation rate than biopsied blastocyst (Goossens et al. 2008; Scott et al. 2013b).

In a recent publication is demonstrated that blastocyst cryopreserved on day 7 have lower but clinically important potential, as in our study (Kovalevsky et al. 2013). For this reason, embryos that not reach blastocyst stage on day 6 should not to be discharged because they may result in an ongoing pregnancy.

Cryopreservation of oocytes, embryos and blastocysts is currently used technique in reproductive medicine (Chhabra and Kutchi 2013; Cobo et al. 2011; Bhattacharya and Kamath 2013). Attempts of using conventional slow-freezing protocols in PGD programs have shown not to be successful, since the lack of the intact zona pellucida protection causes crystal formation that implies extensive damage after thawing. Vitrification protocols, reducing the formation of intracellular ice crystals provides better protection from cryoinjury (AbdelHafez et al. 2010). Different clinical studies have demonstrated both high survival rates of frozen thawed oocytes, embryos and high implantation rates and live births of euploid biopsied blastocysts (Borini et al. 2008). Our study seems also to suggest the harmlessness of all these procedures if carefully performed by a skilled biologist in an IVF lab with quality management system (Practice Committees of ASRM 2013).
